# Obstetrics and Gynecology

**Published:** 1896-02

**Authors:** 


### OBSTETRICS AND GYNECOLOGY.

EDITED BY DR. VIRGIL O. HARDON.

### APPENDICITIS DURING PREGNANCY.

Questions of interest to the profession are constantly arising respecting the occurrence of appendicular inflammation during pregnancy, and the treatment thereof, whether medical or surgical. To country practitioners especially is this a subject of importance, as the exploration of the abdominal cavity necessitates the presence of a competent surgeon, prepared for any emergency. Dr. N. B. Bayley reports a typical case in the New York *Medical Record :*

" An American lady, multipara, was visited when in the sixth month of pregnancy. Her symptoms pointed to an intestinal colic attack, there being looseness of the bowels and marked pain in right side. The usual remedies having been prescribed, the symptoms moderated for a couple of days. Then Dr. Bayley was again sent for, and he found the patient suffering from a combination of symptoms suggesting appendicitis. There was pain over right half of abdomen, with nausea; rigidity of muscular walls over right iliac region, the McBurney point being clearly marked on pressure being applied.

The treatment consisted of administration of morphine, one-sixth grain hypodermically, followed by phenacetin in five-grain doses every three hours. The morphine was later replaced by opium, one grain every three or four hours for two days, and subsequently smaller doses. Calomel was administered in four-grain triturates every three hours, until soreness of gums resulted.

Then potassium chlorite was used as a mouth wash and gargle. The calomel, with occasional doses of Rochelle salts, caused free movements of bowels, and relieved the tympanitic condition. Hot turpentine applications were made externally.

The temperature steadily rose to 103° F. on fourth day. A lessening in rigidity of muscles over iliac region occurred cotemporaneously with decline in temperature. Within a week the temperature was normal, and patient became convalescent. She had a normal, though a more tedious labor than on previous confinements, and made a satisfactory recovery.

With a view to corroborate or strengthen his report of this case, Dr. Bayley furnishes some details of a case of appendicitis with similar symptoms which was under his care at the same time. The second patient was an unmarried woman of twenty-eight years, who had suffered from a previous attack of appendicitis during the year. The prominent symptoms were: Fever rising to 103° F., rigidity of muscles in iliac region, slight dullness, pain at McBurney's point, and somewhat tympanitic abdomen. The treatment was again by opium and calomel, together with ice bag, instead of hot applications of turpentine. Recovery occurred.

---

### PERITONEAL TUBERCULOSIS.

Alexis Thomson, in discussing the cure of this affection by simple abdominal incision, says that the removal of ascitic fluid would seem a satisfactory explanation of the results in cases where a quantity is evacuated, but that it is not the essential factor is shown (1) by the comparative absence of improvement when the fluid is aspirated; (2) by improvement after laparotomy when no fluid is present; and (3) by recoveries when the fluid has been left after operation because encysted. In the peritoneum the tissues and bacilli seem to meet on nearly equal terms and little aid is needed by the tissues. This seems to be furnished by the incision and manipulation rather than by any other factor. Tubercular peritonitis often recovers spontaneously, so always try medical treatment first, careful massage of the abdomen, and restoration of normal intestinal functions. Inunction of ointments probably aids, chiefly by mechanical stimulation. Securing regular and copious evacuations of the bowels by drugs aims at cure of catarrh, cessation of fermentation, and increase in eliminating powers of the mucous membrane. If these fail operate. In diffuse, serous, or purulent ascites incise abdominal wall freely and evacuate fluid; when serous, close at once with iodoform on wound; when purulent, drainage tube two or three days only. When fluid is encysted or circumscribed, operation is especially indicated. When fluid is absent or slight, operation is favorable unless serious tubercular lesions exist elsewhere. Peritoneum must be opened. If incision below umbilicus fails make second or third, these above, as more certain to open the cavity. Never use drainage tube in this condition, on account of danger of intestinal fistula which discharge into the abdominal cavity.

---

### ANTIPYRIN-SALOL IN THE TREATMENT OF UTERINE HEMORRHAGE.

Professor Labadie-Lagrave had used antipyrin successfully in the treatment of certain uterine hemorrhages. It is difficult to introduce the powdered antipyrin into the uterine cavity, so it occurred to him to use antipyrin liquefied with salol, thus producing a medicament at once hemostatic and antiseptic. The following is the mode of procedure: Equal parts of antipyrin and salol are placed in a test-tube so as to occupy about one-third the space; they are then heated over an alcohol lamp, when the mixture is soon transformed into a clear liquid with a slightly brownish tinge.

This is not the time to use the solution, for it will solidify too rapidly. The heating is continued until a well-defined brown color is noticed, when there is no danger of its rapid solidification. The liquid is introduced by means of cotton soaked in it and rolled on a wooden applicator; after seeing that the liquid is not too hot the application is made through the speculum. If the hemorrhage is excessive, two applications are made at the same sitting, after which a tampon soaked in glycerated creosote is placed in the vagina and the patient sent to bed. The applications are free from danger and occasion no pain. Their hemostatic action is rapid, sure, and complete; the hemorrhage is quickly stopped and by the second day there is no trace of hemorrhage; it is rare that the application needs to be repeated. The method is efficacious against hemorrhages due to fungous metritis, to misplacements, fibromyomata, and also to malignant tumors in the beginning, when the hemorrhage is due more to congestion than to ulceration.—*Chicago Medical Reporter.*

---

### Abortion.

Recognizing that any interference with the uterine cavity must be looked upon as a possible source of danger, and, to be free from this danger, must be made aseptically and with antiseptic precautions, Dr. Brooks H. Wells, of New York, strongly urges that, in every case where abortion or miscarriage begins acutely and from natural causes, the ovum be removed by the finger, ovum forceps, or curette, within twenty-four hours after the abortion be considered inevitable, if the entire ovum be not then already expelled, complete expulsion being usually indicated by cessation of pain and hemorrhage. In cases where a portion has been expelled; where there is serious hemorrhage; where the ovum is dead; where there is reason to suspect criminal interference; where there has been continual spotting, foul discharge, or fever, the uterus should be explored and emptied at once, as any delay greatly increases the risk of sepsis. The sharp irrigating curette, followed by gauze drainage, should always be used where there is septic material present or where the endometrium is diseased; in other conditions the finger or a dull instrument is sufficient.—*Medical Record*, September 22, 1894.

---

### Operation in General Suppurative Peritonitis.

The principal reason why surgeons have not succeeded in saving life oftener by operation in these cases is, in the opinion of Miles F. Porter, because the operations have been done too late. He has operated in three cases—two *in extremis*, both of which died, and in one five days after an attempted abortion. As soon as the peritoneum was incised there escaped a large quantity of turbid, stinking serum, followed later by pus of the consistence of cream. There were no adhesions, the pus being free in the peritoneal cavity. This was thoroughly flushed with hot salt solution and drained with glass tube. On second day threatening symptoms necessitated a second flushing. Patient recovered.—*Ex.*

